# ﻿A new species of *Austrocypraea* (Mollusca, Gastropoda, Cypraeidae) from the Pliocene of Flinders Island, Tasmania

**DOI:** 10.3897/zookeys.1123.90917

**Published:** 2022-10-07

**Authors:** Paul C. Southgate, Mike Roberts

**Affiliations:** 1 School of Science, Technology and Engineering, University of the Sunshine Coast, Maroochydore, Queensland 4558, Australia; 2 Australian Centre for Pacific Islands Research, University of the Sunshine Coast, Maroochydore, Queensland 4558, Australia; 3 Post Office, Lady Barron, Flinders Island, Tasmania 7255, Australia

**Keywords:** Cameron Inlet Formation, cowrie, cowry, fossil, taxonomy

## Abstract

A new morphologically distinct species of cowry (family Cypraeidae Rafinesque, 1815) is described from the Pliocene of Flinders Island, Tasmania. *Austrocypraeajimgracei* sp. nov. differs morphologically from other members of the genus and is particularly characterised by the development of a heavily callused labral margin, with a distinct marginal edge that bends up towards the dorsum centrally. This feature is unique within the genus. The new taxon is only the second known *Austrocypraea* from the Pliocene. A revised key to the known *Austrocypraea* fossil species is presented.

## ﻿Introduction

The marine gastropod genus *Austrocypraea* Cossmann, 1903 (Gastropoda, Cypraeidae) is endemic to southern Australia where it has an extensive fossil record from the Oligocene (Yates 2009). Ten of the 12 currently known *Austrocypraea* fossil species are from the Miocene: *A.archeri* (Tenison-Woods, 1876), from the Early Miocene (Longfordian) of Tasmania; *A.contusa* (McCoy, 1877), *A.scalena* (Tate, 1890), *A.subsidua* (Tate, 1890), *A.ampullacea* (Tate, 1890), *A.parallela* (Tate, 1890), *A.constricta* Schilder, 1935, *A.subcontusa* Schilder, 1935, and *A.goudeyana* Fehse, 2013, from the Middle Miocene (Balcombian) of Victoria; and *A.rumballi* Fehse, 2003 from the Middle Miocene (Balcombian) of South Australia. The youngest member of the genus, *A.amae*Fehse and Kendrick 2000, is the only known Pliocene species. *Austrocypraeaamae* is generally acknowledged to be ancestral to the only extant member of the genus, *A.reevei* (Sowerby II, 1832), which lives on reefs associated with sponges, from central Western Australia to South Australia (Wilson 1993; Lorenz 2017).

The geology and fossil molluscan fauna of Flinders Island, in the Bass Strait off the north-eastern coast of Tasmania, were described by Sutherland and Kershaw (1970). The Pliocene Cameron Inlet Formation of Flinders Island is relatively rich in mollusc fossils, and was recognised by Darragh (1985) (as ‘Molluscan Assemblage XVII - Flinders Island’) as one of 18 informal assemblages characterising molluscan biogeography and biostratigraphy of the Tertiary of south-eastern Australia. Mollusc fossils from the Cameron Inlet Formation are generally obtained from spoil material excavated for drainage channels and farm dams, and four members of the Cypraeidae (cowries) have so far been reported: *Umbiliahestitata* (Iredale, 1916), *U.furneauxensis* Southgate, Militz & Roberts, 2021, *Notocypraeajonesiana* (Tate, 1890), and *N.angustata* (Gmelin, 1791) (Sutherland and Kershaw 1970; Darragh 1985; Goudey 2015; Southgate et al. 2021). A number of specimens of an apparently undescribed species of fossil cowry were recently recovered from excavated material in the Lackrana area of Flinders Island. Described here as *Austrocypraeajimgracei* sp. nov., it is the second representative of the genus from the Pliocene.

## ﻿Materials and methods

### ﻿Examined material

All examined specimens were recovered from material excavated for farm dams in the Lackrana area of Flinders Island, Tasmania. Assignment of specimens to the late Pliocene Cameron Inlet Formation was confirmed by reference to molluscan assemblages previously described for the Cameron Inlet Formation and the Pleistocene Memana Formation that disconformably overlies the Cameron Inlet Formation (Sutherland and Kershaw 1970; Darragh 1985).

### ﻿Morphological methods

Shell length (**L**), width (**W**), and height (**H**) were measured as described by Lorenz (2017) using a vernier calliper. Counts of columellar teeth (**CT**) excluded the terminal ridge bordering the anterior canal but included the posterior-most denticle that merges with the anterior edge of the columella callus bordering the posterior canal. All labral teeth (LT) were counted. Quantitative comparisons used the shell formula [L (W/L-H/L-H/W) nLT: nCT], where L = average shell length (mm), W/L = average width/length ratio (%), H/L = average height/length ratio (%), H/W = average height/width ratio (%), and nLT and nCT are normalised labral and columellar tooth counts, respectively, for a hypothetical shell length of 25 mm (Schilder 1935), calculated as described by Lorenz (2017). Descriptive terminology generally follows that of Lorenz (2002, 2017).

### ﻿Abbreviations

**TMAG**Tasmanian Museum and Art Gallery, Hobart, Australia;

**AM**Australian Museum, Sydney, Australia;

**MR** Mike Roberts collection, Flinders Island, Tasmania, Australia;

**PS** Paul Southgate collection, Brisbane, Australia.

## ﻿Results

### ﻿Systematics

**Class Gastropoda Cuvier, 1795**

**Order Littorinimorpha Golikov & Starobogatov, 1975**

**Superfamily Cypraeoidea Rafinesque, 1815**

#### Family Cypraeidae Rafinesque, 1815

##### ﻿Genus Austrocypraea

Taxon classificationAnimaliaLittorinimorphaCypraeidae

Cossmann, 1903

9C78BBCB-16A7-54DC-BD95-70B3C26BCA86

###### Type species.

*Cypraeacontusa* McCoy, 1877, by original designation. Balcombian, Middle Miocene, Fossil Beach, Victoria, Australia.

##### ﻿ Austrocypraea jimgracei sp. nov.

Taxon classificationAnimaliaLittorinimorphaCypraeidae

6C60B3A1-CA73-5D00-B409-C3A5C7128B42

https://zoobank.org/C1F042E5-6E4B-4038-A757-53B289C377CD

**Figure 1.**
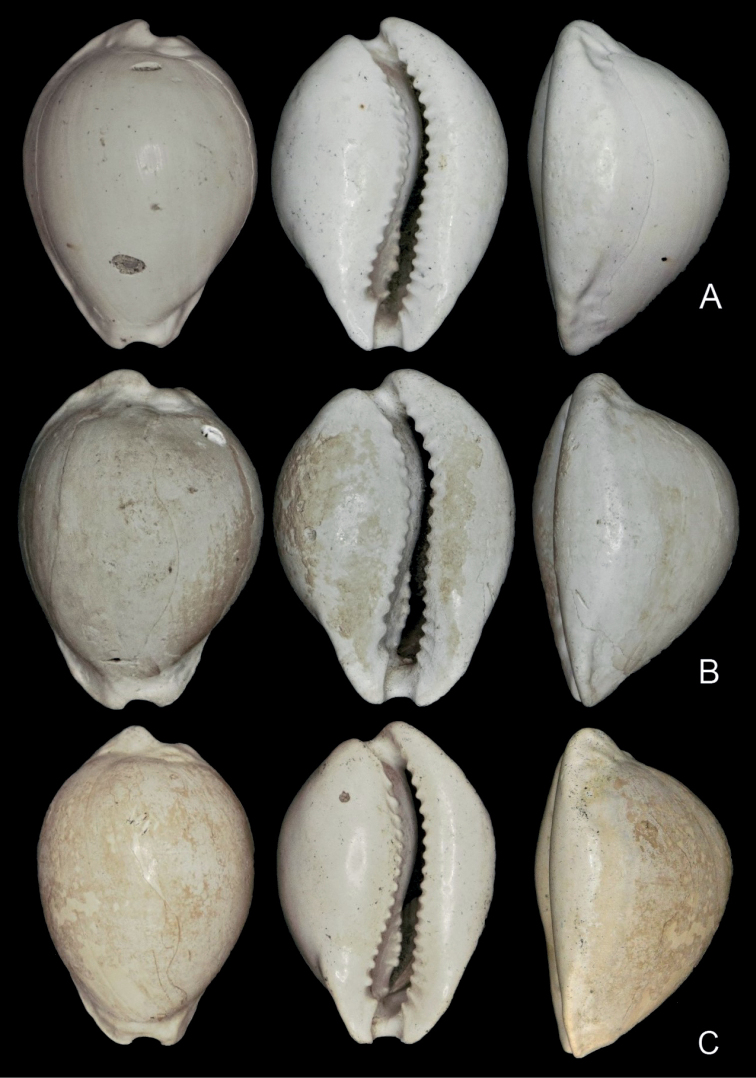
*Austrocypraeajimgracei* sp. nov.; dorsal, ventral and marginal (labral) aspects **A** holotype, TMAGZ10628 **B** paratype 1, TMAG Z10629 **C** paratype 5, senior author collection (PS CF.174)

**Figure 2.**
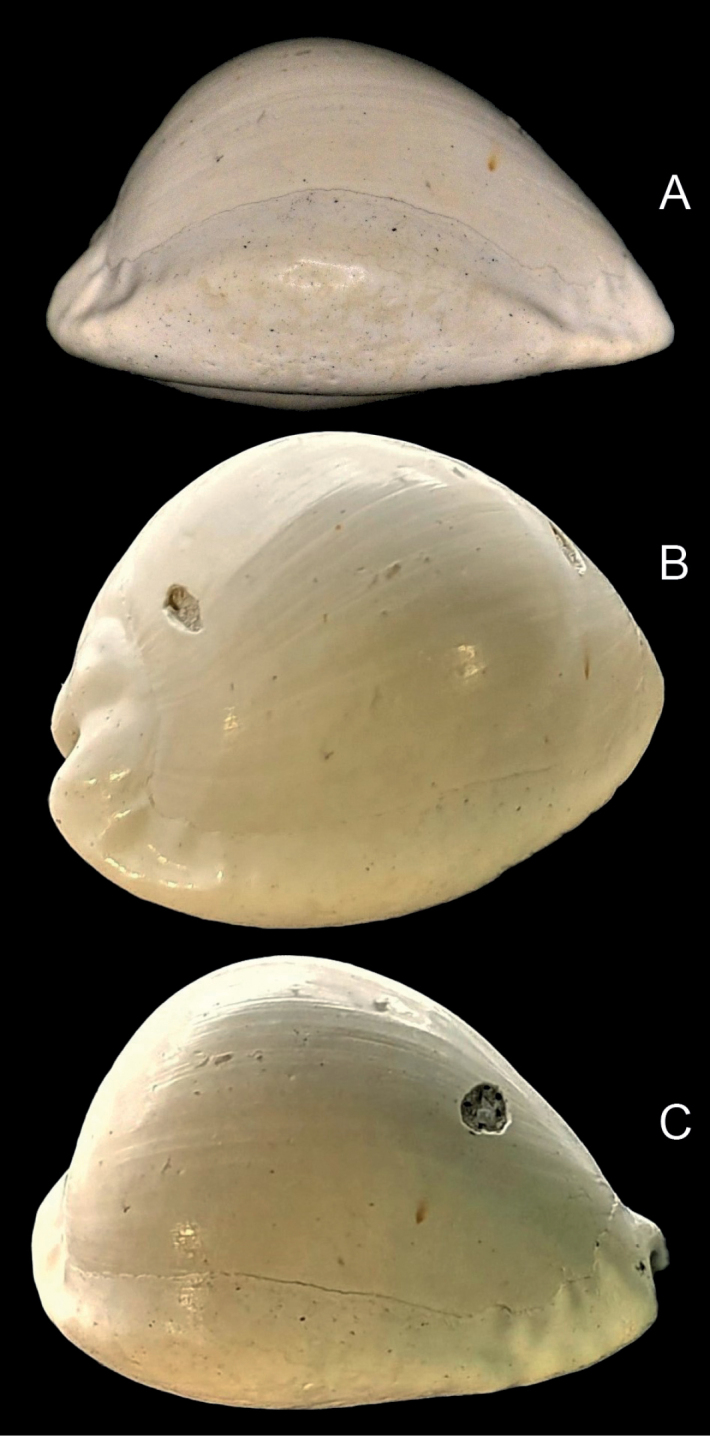
Detail of labral margin of the holotype of *Austrocypraeajimgracei* sp. nov. (TMAGZ10628) showing labral margin (**A**), posterior labral groove (**B**) and anterior labral groove (**C**).

**Figure 3.**
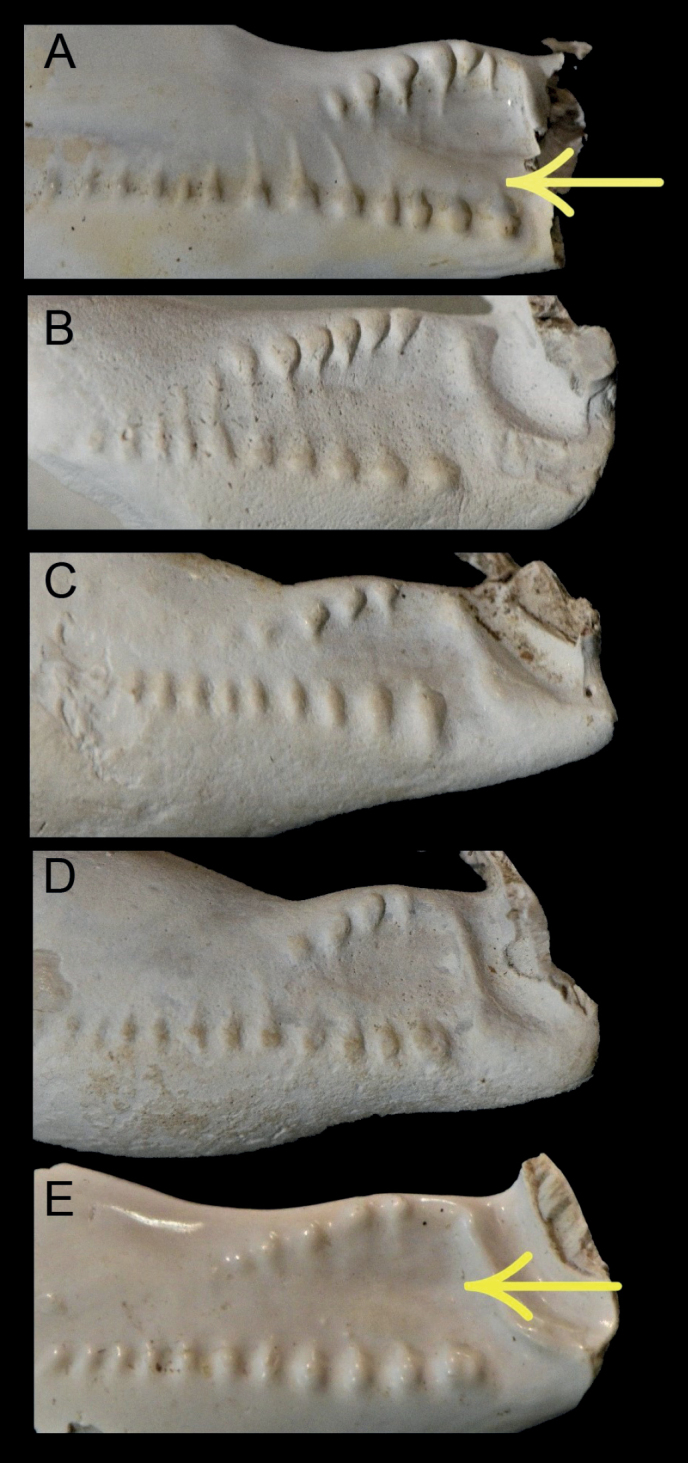
Detail of the fossula of sectioned shells of *Austrocypraeajimgracei* sp. nov., showing variation amongst specimens (TMAGZ10630). Arrows indicate a slightly raised ridge (**A**) and shallow longitudinal depression (**E**) sometimes present on the fossula.

Figs 1 , 2 , 3 , Table 1

###### Material examined.

***Holotype*.** Australia, Lackrana, Flinders Island, Tasmania; 40°06'37"S, 148°10'18"E; May, 2012; P.C. Southgate and M. Roberts leg.; dry specimen (fossil); among spoil material excavated for farm dam; TMAGZ10628.

***Paratypes*.** Australia; same location as holotype; May, 2012-Feb, 2021; P.C. Southgate and M. Roberts leg.; dry specimens (fossils); among spoil material excavated for farm dam; TMAGZ10629 (1 specimen); AM F.156043 (1 specimen); AM F.156044 (1 specimen); MR 635 (1 specimen); PS CF.174/175 (2 specimens).

###### Other material.

Australia; same location as holotype; May, 2012-Feb, 2021; P.C. Southgate and M. Roberts leg.; dry specimens (fossils); among spoil material excavated for farm dam; PS CF.305/306 (2 specimens); MR 657/658 (2 specimens); TMAGZ10630 (five partial specimens).

###### Diagnosis.

*Austrocypraeajimgracei* sp. nov. can be separated from all other members of the genus, fossil and extant, by a combination of the following characteristics: shell ovate to sub-pyriform, humped, highest point towards posterior, shell height around 71% length, shell width around 59% length; anterior extremity subtruncate, not extended in lateral profile, supported by well-defined anterior lateral flanges; protoconch paucispiral, spire projecting, overlain by callus. Aperture gently curved to the left posteriorly, widening slightly towards anterior; evenly spaced, relatively strong dentition; 13–17 columellar teeth, restricted to aperture, larger towards anterior; 17–21 labral teeth are longer, incised, and restricted to aperture margin. Fossula is broad, concave and smooth centrally, with shallow, barely discernible longitudinal depression or slightly raised ridge sometimes present; fossular margin with 4–6 denticles, visible in ventral view; anterior denticles not linked to adjacent columellar teeth by transverse ridges, but fine ridges may link posterior fossular denticles to adjacent columellar teeth. Columella smooth posterior to the fossula, lacking a defined columella ridge. Labral margin heavily callused, forming a distinct marginal edge, bent up towards the dorsum centrally; shallow anterior and posterior labral grooves may accommodate small, irregular, often elongate, pustules dorsal to the labral marginal edge; marginal edge may be weakly crenulate where marginal pustules intersect.

###### Description.

Average size for the genus (Table 1); shell length 23.1–25.5 mm (mean 24.5), W/L = 69–73% (mean 71%), H/L = 58–61% (mean 59%), H/W = 82–85% (mean 84%); ovoid to sub-pyriform, maximum shell height towards posterior; protoconch paucispiral, rounded; spire projecting, overlain by callus (Fig. 1). Shell formula: [24 (71-59-84) 19:15]. Dorsal surface smooth except for weak longitudinal growth lines. Basal callus strongly developed; base rounded. Shell margins callused; left margin rounded, smooth; labral marginal callus well developed, with distinct marginal edge, bent up towards the dorsum centrally (Fig. 2A); more sharply margined anteriorly and posteriorly, forming shallow labral grooves that may accommodate pustules or small tubercules, often elongate, dorsal to the marginal edge (Fig. 2B, C). The right marginal edge may be slightly crenulate where pustules intersect. Anterior and posterior canals are deep and bordered by strong projecting callus; anterior canal with dorsoventral orientation, not angled; anterior terminal subtruncate, hardly extended, supported by anterior lateral flanges; anterior tips moderately pointed. Posterior canal short, bent to the left; bordered on the left by well-developed columellar callus, not extending as far as the posterior end of the labrum. Aperture gently curved to the left posteriorly, widening anteriorly. Fossula broad (Fig. 3), concave and constricted posteriorly; bordered anteriorly by a well-defined terminal pleat, thickening where it joins the terminal ridge. Fossula margin with 4–6 denticles becoming weaker posteriorly; anterior denticles not linked to adjacent columellar teeth by transverse ridges; poorly developed ribs may link the smaller denticles at the posterior end of the fossula with collumellar teeth, where the fossula merges with the columella (Fig. 3B). Very weak ridges may extend onto the fossula from the marginal denticles and from the anterior-most columellar teeth, but they do not join; central fossula smooth, with shallow, longitudinal depression (Fig. 3E) or slightly raised ridge on the central fossula sometimes evident (Fig. 3A). A well-defined fossular gap is present at the anterior end of the fossula margin, between the anterior-most marginal denticle and the dorsal extremity of the terminal pleat. Columella lacks a defined columellar ridge and is smooth posterior to the fossula. Columellar teeth (13–17, Table 1) spaced about one tooth width apart; present along the length of the columella, terminating at the anterior edge of columella callus bordering the posterior canal; anterior columella teeth stronger, first tooth generally stronger than those posterior to it, and separated from the terminal ridge by a prominent anterior gap. Labral teeth (17–21, Table 1) restricted to aperture, longer anteriorly, more elongate and more numerous than columella teeth.

**Table 1.** Descriptions and repositories of the type series of *Austrocypraeajimgracei* sp. nov.

Specimens (repository)	Length (mm)	Width (mm)	Height (mm)	Columellar teeth	Labral Teeth
Holotype (TMAGZ10628)	24.2	17.5	14.5	16	21
Paratype 1 (TMAG Z10629)	25.4	18.5	15.4	13	17
Paratype 2 (AM F.156043)	23.1	16.4	13.8	16	20
Paratype 3 (AM F.156044)	25.2	18.2	14.9	16	18
Paratype 4 (MR 635)	23.6	16.4	13.9	14	18
Paratype 5 (PS CF.174)	24.4	16.9	14.4	15	20
Paratype 6 (PS CF.175)	25.5	17.7	14.9	17	20
Mean (± SD)	24.5 (±0.9)	17.4 (±0.8)	14.5 (±0.6)	15.3 (±1.4)	19.0 (±1.5)

***Variation*.** Available specimens show variation in the angle of slope of the fossula, the number and form of fossular marginal denticles, and the presence, or otherwise, of a shallow depression or a slightly raised ridge on the central part of the fossula (Fig. 3). Pustules dorsal to the anterior and posterior labral margins may or may not be present or visible, and this probably relates to specimen maturity and the degree of preservation. Pustules may produce a weakly crenulate marginal edge in some specimens where (and if) they intersect. This feature is present in the holotype and its development is likely related to specimen maturity and the degree of callus development.

###### Differential diagnosis.

Shell shape in cowries is commonly expressed using a ‘shell formula’ which reports linear shell measurements, and their ratios, as well as normalised tooth counts (Bridges and Lorenz 2013). The shell formula of *Austrocypraeajimgracei* sp. nov. is compared with those of all other *Austrocypraea* fossil species, for which morphometric data are available, in Table 2. In terms of shell shape (i.e., W/L, H/L and H/W), *A.jimgracei* sp. nov. is closest to *A.amae* and *A.rumballi* (Table 2) and a broader range of shell characteristics is compared for these three species in Table 3. The new species is similar in size and dimensions to *A.amae*, where shell width relative to length (71%), height relative to length (60%), and height relative to width (84%) are very similar to values of 71%, 59% and 84%, respectively, for the same parameters in *A.jimgracei* sp. nov. (Table 2). The anterior extremity of *A.amae* is shorter and less produced, and the anterior canal is wider, deeper and more angled than in *A.jimgracei* sp. nov. The anterior lateral flanges supporting the anterior extremity of *A.jimgracei* sp. nov. are more developed than in *A.amae*. The posterior extremity is more produced in *A.jimgracei* sp. nov. than in *A.amae*, particularly on the right side. The new species can be easily separated from *A.rumballi* by its much larger size.

**Table 2.** Shell formulae [L (W/L – H/L – H/W) nLT: nCT] for known *Austrocypraea* fossil species for which complete morphometric data are available.

Species	Shell formula	Data source(s)
*Austrocypraeaconstricta*	14 (59-48-81) 26:14	Schilder (1935)
*A.archeri*	21 (61-50-86) 22:17	PS collection
*A.subsidua*	22 (65-52-80) 25:18	Schilder (1935)
*A.scalena*	30 (64-57-89) 24:16	Schilder (1935)
*A.subcontusa*	15 (67-55-82) 18:15	Schilder (1935)
*A.contusa*	26 (74-66-89) 21:17	Schilder (1935)
*A.ampullacea*	34 (56-53-94) 27:22	Schilder (1935)
*A.parallela*	18 (53-46-86) 32:20	Schilder (1935)
*A.amae*	28 (71-60-84) 22:16	Fehse and Kendrick (2000)
*A.rumballi*	16 (73-63-85) 16:14	Fehse (2003); Yates (2008)
*A.goudeyana*	22 (68-58-86) 17:16	Fehse (2013)
*A.jimgracei* sp. nov.	24 (71-59-84) 19:15	This study

Clear differences in fossula structure also separate *A.jimgracei* sp. nov. from *A.amae* and *A.rumballi*. The fossula of *A.amae* is crossed by ribs which are continuous with the anterior columellar teeth and extend to the inner margin of the fossula (Table 3). Denticles on the inner fossular margin of *A.jimgracei* sp. nov. are separated from adjacent anterior columellar teeth by a smooth central area of the fossula. The fossula of *A.rumballi* is similar to that of *A.jimgracei* sp. nov. but differs by protruding further into the aperture, having a greater number of denticles on the inner margin (generally 6–7), and transverse ridges that link the denticles to columellar teeth, at least posteriorly (Yates 2008). An interesting feature of the fossula of *A.rumballi* is an indistinct tubercle or longitudinal ridge in the middle of the fossula between the terminal ridge and the first or second transverse ridge of the fossula (Yates 2008). A shallow longitudinal depression or slightly raised ridge is sometimes present in the middle of the fossula of *A.jimgracei* sp. nov. (Fig. 3); however, the form of this feature varies and it is absent in some specimens.

**Table 3.** Comparison of shell characters of *Austrocypraeaamae*Fehse and Kendrick 2000, *A.rumballi* Fehse, 2003 and *A.jimgracei* sp. nov.

Character	Species		
	*Austrocypraeaamae*	*A.rumballi*	*A.jimgracei* sp. nov.
Length (mm):	20.7–38.9	11.0–22.1	23.1–25.5
Shape:	ovate, sub-pyriform to sub-cylindrical; highest point in posterior third. Short anterior extremity not extended in lateral profile.	broadly ovoid; inflated specimens globular. Anterior extremity short but extended in lateral profile.	ovate to sub-pyriform; highest point towards posterior; short anterior extremity, not extended in lateral profile, supported by well-developed lateral flanges.
Sculpture:	moderately to weakly malleate, mainly posteriorly; base smooth.	malleation present on the left side of many specimens but absent in others; base smooth.	malleation absent; base smooth.
Fossula:	broad, impressed, traversed by 6 or 7 ribs continuous with columella teeth.	broad with projecting inner margin; fossula denticles (~ 6) linked to columella teeth by transverse ridges becoming obsolete anteriorly. Indistinct tubercle or ridge centrally.	broad, inner margin with 4–6 denticles that do not connect to columella teeth; central fossula smooth. Shallow, longitudinal depression or raised area sometimes present centrally.
Labral margin:	rounded but not heavily thickened; generally rounded in posterior profile.	rounded but not heavily thickened; generally rounded in posterior profile.	heavily callused, forming a distinct marginal edge, bent up centrally; pustules or small tubercules often present dorsal to marginal edge.
Data source(s):	Fehse and Kendrick 2000	Fehse 2003; Yates (2008)	This study

The Miocene species *A.contusa* and *A.goudeyana* have similar size and proportions to *A.jimgracei* sp. nov., and the form of the anterior extremity, supported by distinct anterior lateral flanges, may also be similar for these species. However, *A.contusa* and *A.goudeyana* are readily separated from *A.jimgracei* sp. nov. because of their more produced anterior extremity, narrower aperture, which has consistent width throughout, and stronger dentition that may extend onto the columella. The form of the heavily callused labral margin of *A.jimgracei* sp. nov., that is bent up towards the dorsum centrally, is unique within the genus. Other species within the genus, such as *A.contusa*, *A.goudeyana* and *A.subcontusa*, may also develop a well-defined thickened labral margin, but unlike that of *A.jimgracei* sp. nov., when present, it generally forms a thin step-like rim to the shell margin that does not bend up towards the dorsum. The shells of a number of *Austrocypraea* species, including the extant *A.reevei*, have shallow contusions or ‘malleation’ on the dorsal surface of the shell, but this is not a ubiquitous feature of the genus. For example, malleation is prominent on the shells of *A.contusa* and *A.goudeyana*, less prominent and generally restricted to the posterior half of the body whorl in *A.rumballi* and *A.amae*, obscure or absent in *A.scalena*, but totally lacking in *A.onkastoma*, *A.archeri*, *A.subsidua* and *A.parallela*, and in all examined specimens of *A.jimgracei* sp. nov.

###### Etymology.

Named in honour and in memory of the late Jim Grace of Lackrana, Flinders Island, on whose property all specimens of the new species were recovered.

###### Distribution.

Known only from the Cameron Inlet Formation, Lackrana area, Flinders Island, Tasmania.

### ﻿Key to the known fossil species of Austrocypraea

The following key is based on shell morphology and is modified from Schilder (1935) to include subsequently described species.

**Table:** 

1	Shell ovate, globular or pyriform; fossula abruptly constricted posteriorly	**2**
–	Shell cylindrical; fossula scarcely constricted posteriorly, broad but rather shallow	***A.parallela***
2	Fossula very broad, concave, projecting; dorsum smooth, small contusions more apparent posteriorly if present; columella smooth posteriorly	**3**
–	Fossula rather narrow, concave; dorsum with numerous, close small contusions throughout; columellar teeth often produced across the columella posteriorly	**5**
3	Shell not exceeding 17 mm; aperture sinuous, inner lip rather constricted in the anterior third; shell subcylindrical	***A.constricta***
–	Shell exceeding 17 mm	**4**
4	Base flattened; aperture rather wide; anterior top of outer lip rather rounded; fossula extremely broad, irregularly ribbed	***A.subsidua***
–	Base convex; aperture equally narrow but may widen slightly anteriorly	**5**
5	Shell generally not exceeding 20 mm	**6**
–	Shell exceeding 20 mm	**7**
6	Shell ovate, slightly depressed; dentition rather course, columella teeth often produced	***A.subcontusa***
–	Shell ovate to globose; columella teeth short, second tooth weakly developed; fossula greatly protruding, with central tubercule	***A.rumballi***
7	Elongate; dentition extremely fine and numerous	***A.ampullacea***
–	Shell inflated, pyriform, ovate or globular	**8**
8	Pyriform to globular, inflated, dorsum intensely malleate	**9**
–	Ovate to sub-pyriform, inflated, dorsal contusions reduced or absent	**10**
9	Anterior terminal collar elongated, elevated in lateral profile	***A.goudeyana***
–	Anterior terminal collar shorter and low in lateral profile	***A.contusa***
10	Fossula ribbed, or marginal denticles present	**11**
–	Fossula smooth, without marginal denticles; columellar teeth developed posteriorly, no dorsal contusions	***A.onkastoma***
11	Aperture equally narrow throughout	**12**
–	Aperture widening towards anterior	**13**
12	Sub-pyriform; narrow, ribbed fossula; no dorsal contusions; rarely exceeding 27 mm	***A.archeri***
–	Ovate, mostly exceeding 27 mm; fossula regularly ribbed	***A.scalena***
13	Ovate to sub-pyriform; fossula traversed by 6 or 7 ribs continuous with columellar teeth; contusions, if present, more apparent posteriorly	***A.amae***
–	Ovate to sub-pyriform; fossula smooth centrally, margin with 4–6 denticles; no dorsal contusions; well-developed labral margin	***A.jimgracei* sp. nov.**

## ﻿Discussion

*Austrocypraeajimgracei* sp. nov. is only the second known member of the genus from the Pliocene and this description increases the number of known fossil species within *Austrocypraea* to thirteen. The new species has characteristics that are typical of the genus, including a well-produced denticulate fossula, a paucispiral protoconch indicating intracapsular development, and a projecting spire. It also has some characteristics that are not present in any other *Austrocypraea* species, including a heavily callused labral margin and the presence of pustules or small tubercules within the labral grooves above (dorsal to) the marginal edge. Extensive molecular analysis within the family Cypraeidae (e.g., Meyers 2003, 2004) has shown that the closest living relatives to *Austrocypraea* are within the genus *Raybaudia* Lorenz, 2017, and that both genera evolved from *Lyncina* Troschel, 1863, shortly after it split from *Callistocypraea* Schilder, 1927 (Lorenz 2017). It is interesting to note that species with similar development of the labral shell margin, some of which may be tuberculate or pustulate, are present within these three genera most closely related to *Austrocypraea*. It is also interesting to note that despite the close phylogenetic relationship between these three Indo-West Pacific genera and *Austrocypraea*, they differ from *Austrocypraea* in undergoing pelagic not intracapsular development (Lorenz 2017).

## Supplementary Material

XML Treatment for Austrocypraea

XML Treatment for Austrocypraea jimgracei
